# Challenges in the Care of Cancer Patients in Emergency Departments

**DOI:** 10.3390/cancers18020231

**Published:** 2026-01-12

**Authors:** Thomas Licht

**Affiliations:** 1 Ludwig Boltzmann Institute for Rehabilitation Research, Reizenpfenniggasse 1, 1140 Vienna, Austria; thomas.licht@lbg.ac.at; 2 Onkologisches Rehabilitationszentrum St. Veit im Pongau, St. Veiter Str. 48, 5621 St. Veit im Pongau, Austria

Cancer patients often suffer complications from their underlying disease or from adverse effects of their oncologic therapies. They are therefore admitted to the emergency room more frequently than the general population. Among the many reasons for treatment in emergency departments (EDs), the most frequent causes are abdominal or chest pain, dyspnea, fever, and nausea or vomiting [1]. In this setting, cancer patients are treated by emergency medicine (EM) physicians, whose procedures and concepts may differ from those of the oncologists who previously treated these patients. A review of expert opinions concluded that the current state of acute oncology education and the training of EM physicians is insufficient [2]. It is conceivable that this might lead to misunderstandings between EM physicians and oncologists, as well as differing priorities or inconsistent treatment strategies.

To resolve the difficulties this causes for patients and for the medical staff caring for them, it is important to understand the different approaches and views. In their article, entitled “Perceived Gaps in Oncologic Emergency Care for Patients with Cancer: A Qualitative Comparison of Emergency Medicine and Oncologist Physician Perspectives”, published in the section Cancer Survivorship and Quality of Life, M.K. Wattana and collaborators investigate potential gaps in emergency care for cancer patients from the perspective of both groups of physicians [3]. Physicians from five medical centers in the USA. participated in this survey. They described three main themes from the perspective of both groups of physicians, namely system-based constraints; direct patient-care-related issues; and knowledge gaps. Whereas EM physicians identified knowledge gaps in cancer therapeutics and gaps in general oncologic emergencies as the most pertinent issues in this study, the oncologists recognized long delays in care, variability in care, and communication issues between EM physicians and oncologists as the most relevant concerns. Furthermore, the three next most common issues for ED physicians were physician comfort level, the timing or location of an initial goal of care discussion, and challenges with the follow-up process. In addition, various other issues were perceived by either group including, but not limited to, the overcrowding of ED, appropriate disposition, communication between ED physicians and oncologists, and care expectations and prognostication. Notably, oncologists specified far more perceived knowledge gaps than ED physicians. Besides gaps in knowledge of analgesics, general emergencies and cancer therapeutics, which were also recognized by EM physicians, they pointed to knowledge gaps in general oncology, neutropenic patients, patient acuity, surgical complications, transfusion medicine, therapeutics and treatment adverse effects, neuro-oncologic emergencies, and other areas.

Difficulties in caring for patients with a suspected cancer diagnosis in emergency departments (ED) are also highlighted in a recent study from centers in Ontario, Canada (4). Four major themes could be identified from the perspective of ED physicians in this study: challenges in the broader healthcare system that create systemic gaps and structural barriers; institutional and professional limitations shaping the EM physicians’ experience of managing cancer patients; patient-level factors and experiences with receiving a cancer diagnosis in the EM; and concerns about poorly coordinated care from the emergency department. Of note, some views were particularly negative in this study, e.g., a quote of an ED physician who believed that “the emergency department has become a catch-all or, some people think, the garbage can of our healthcare system, and everything that no one else can find out what to do with, they send them to us” [4]. Such statements should serve as a reminder that cancer patients are among the groups that can be stigmatized in healthcare facilities, which was shown by a meta-analysis [5].

The problems identified in EM may even have a negative impact on the prognosis of cancer patients. In a British study, patients who presented as emergency cases were found to have higher short-term mortality in all cancer types compared with non-emergency patients [6]. Moreover, a large cohort study revealed that patients who had been to EDs prior to their respective cancer diagnosis had statistically significantly higher risk of mortality, compared with matched patients without ED use [7]. Further evidence for challenges with cancer patients in EM comes from a systematic review [8]. The authors found that patients diagnosed with cancer within the ED presented at more advanced stages and had poorer survival rates than patients diagnosed with cancer outside of the emergency department. This finding was upheld irrespective of the type of cancer and confounding factors.

Many patients are admitted to the ED, even though they can be treated outside. In addition to patient-related issues, this may reduce overcrowding and costs and improve the quality of care. Hence, efforts have been undertaken to identify oncology patients at high risk for potentially preventable ED visits [9]. In this investigation, preventable visits were most common among stage I to III breast cancer patients and those undergoing systemic therapy. Conversely, stage IV disease, with either lung or gastrointestinal carcinomas, and shorter distances to the ED proved to be predictors of non-avoidable visits.

From the perspective of ED physicians, the most relevant gaps were related to insufficient knowledge of oncologic therapies and emergencies [3]. A lack of knowledge in oncology, particularly with respect to the efficacy and potential adverse effects of new anticancer drugs, can lead to treatments that do not adhere to clinical practice guidelines. This results in inconsistent outcomes or, in the worst cases, even malpractice. The ED physicians indicate an urgent need for oncology education and training. The author of the current research paper in *Cancers* has already developed an oncologic curriculum that helps prepare EM residents to recognize and manage the most common oncologic emergencies [10]. For reference, multiple resources are available in the form of evidence-based guidelines from several professional organizations including the National Comprehensive Cancer Network (NCCN), the American Society of Clinical Oncology (ASCO), and the European Society of Medical Oncology (ESMO). In addition, comprehensive review articles on oncologic emergencies are also available [11].

The need for the improvement of emergency care of cancer patients in EDs has been recognized. In 2015, the National Cancer Institute conducted a workshop to identify research priorities and appropriate resources [12]. Thereby, infrastructure was proposed to address the emergency care of patients with cancer. As a result, the Comprehensive Oncologic Emergencies Research Network (CONCERN) consortium was established. Research from this study group contributed to better understanding the reasons why cancer patients are admitted to EDs [1]. Different models for optimization of care for cancer patients in EDs are being developed, e.g., creation of cancer-specific EDs or oncology urgent care clinics, respectively, in medical centers with appropriate resources [13,14]. Such institutions offer specialized care and have been shown to reduce the use of EDs by cancer patients [14].

The emergency treatment of cancer patients by EM physicians in the ED presents difficulties that can lead to poorer outcomes than in specialized oncology facilities. It is therefore essential to identify the challenges that remain unresolved from the perspective of both EM physicians and oncologists, who routinely treat these patients. From the perspective of EM physicians, one primary concern is related to gaps in their knowledge regarding oncological therapies and the complications associated with them. Oncologists describe long delays in care, variations in care, and communication problems. Identifying gaps in emergency care can help improve the treatment of oncology patients in the ED. To this end, research programs and special training units for EM physicians have been established. Further improvements are expected through patient care in cancer-specific EDs or oncology urgent care clinics.
